# Just a story? Leadership, lived experience and integrated care

**DOI:** 10.1111/hex.14084

**Published:** 2024-05-21

**Authors:** Robin Miller, Nieves Ehrenberg, Caroline Jackson, Viktoria Stein, Wilma Van der Vlegel‐Brouwer, Anne Wojtak

**Affiliations:** ^1^ School of Social Policy University of Birmingham Birmingham UK; ^2^ VM Partners Integrating Health and Care Lisbon Portugal; ^3^ Department for Public Health & Primary Care Leiden University Health Campus The Hague Netherlands; ^4^ SevenSenses Institute Nieuwegein The Netherlands; ^5^ Dalla Lana School of Public Health University of Toronto Toronto Canada

**Keywords:** citizen leaders, integrated care, leadership, stories, storytelling

## Abstract

**Background:**

Integrated care is based around values of involvement and shared decision‐making, but these are not often reflected within planning and implementation. Barriers include continued emphasis on professional and managerial perspectives, skills gaps on how best to engage people and communities and insufficient investment in involvement infrastructure. Despite such challenges, people with lived experience have still led changes in policy and services.

**Design:**

Qualitative study involving 25 participants with lived experience from 12 countries. Participants shared their background stories and engaged in semistructured interviews relating to leadership identity, experience of influencing and personal learning. Transcripts were analysed through a framework approach informed by narrative principles.

**Results:**

Participants were motivated by their own experiences and a wish to improve care for future individuals and communities. Sharing their story was often the entry point for such influencing. Participants gained skills and confidence in story telling despite a lack of support and development. Many felt comfortable being described as a leader while others rejected this identity and preferred a different title. No common alternative term to leader was identified. Influencing services required considerable personal cost but also led to new networks, skills development and satisfaction when change was achieved.

**Discussion:**

Leadership within integrated care is often awarded to those with structural power related to management or clinical seniority. People with lived experience are though uniquely placed to identify what needs to change and can develop inspiring visions based around their personal stories. Claiming identity as leader can be challenging due to traditional notions of who is eligible to lead and unwillingness by professionals and managers to grant such identity.

**Conclusions:**

People with lived experience should be recognised as leaders of integrated care and have access to developmental opportunities and practical support to strengthen their skills, including that of storytelling.

**Patient and Public Contribution:**

The research was instigated on the request of a community advisory board of people with lived experience who shaped its design, contributed to the analysis and informed the conclusions and implications.

## 1 INTRODUCTION

Leadership has been long recognised as an important enabler of innovation and quality in health and social care.^1^ It influences organisational and professional practice including team cultures, staff motivation, interprofessional collaboration and receptivity to new ways of working.^2^, ^3^ While positive and engaged leadership can lead to better outcomes, its absence (or indeed presence of toxic leadership) can result in a decline in the quality of service and negatively affect wellbeing of staff.^4^, ^5^, ^6^ Leadership is interpreted in this article as ‘a process whereby an individual influences a group of individuals to achieve a common goal’.^7^ ^,p.6^ It is conceptualised as a dynamic interaction in which leaders and followers both have active roles, and in which leadership behaviour adapts to differing contexts.^8^ While leaders have traditionally been seen as those in senior political, faith and management roles, leadership is demonstrated by other actors in society. In health care, the importance of clinical leadership to standards of practice is well established.^9^ Leadership makes a vital contribution within integrated care through setting out the case for change, encouraging professionals to adopt new collaborative practices and challenging organisations to share resources.^8^, ^10^

Values of involvement and shared decision making are central to integration including within strategic decision making on investment and service design.^11^ The responsibility of embedding these values is often laid at the door of senior leaders.^12^ While those with structural power should contribute, there is a danger that people with lived experience are seen as passive participants whose involvement is decided by others.^13^ People with lived experience within this article are defined as those who have insights from accessing health and social care services including those with a disability, long‐term condition and who are family carers. Such passivity has been rejected by people themselves, who have instead taken up active roles in shaping what and how they influence.^14^ In doing so they are demonstrating citizen leaders who ‘have power … and [use this to] take actions for the benefits of other citizens’.^15^ ^,p.4^ They are ‘changing the rules of the game, to empower non‐powerful others to have an effective voice or role in solving public problems’.^16^ ^,p.49^ Similarly, within research, there are calls for people with lived experience to go beyond commenting on researcher defined questions and methodologies but rather set the overall purpose and focus of what is studied.^17^

A common tool used by leaders within organisations and politics are stories.^18^, ^19^ These can illuminate challenges and build community through creating empathy and facilitating coherence.^20^ This reflects how storytelling within human society facilitates collective meaning and maintains cultural norms and social structures between generations.^21^, ^22^ Leaders use narratives to build their personal authenticity and credibility to potential followers.^23^ A well‐told story engages listeners with their own experiences and emotions and facilitates new insights on their individual purpose and identity.^24^ Stories are closely connected to the core leadership tasks of framing and sensemaking and will adapt over time to reflect changing contexts and the interests of an audience.^25^, ^26^ Consequently, skills of creating and delivering a story are now common components of leadership development programmes.^27^ Health care has traditionally been based on evidence based on a narrow set of quantitative research methods, but stories are now recognised as providing robust insights which can directly inform improvement towards person centred and coordinated care.^27^, ^28^, ^29^ Stories can strengthen research through contextualising experience, practically illustrating concepts and providing greater depth and shared understanding.^30^ Open digital libraries are being curated to provide a repository for people's stories which can be accessed by those facing similar situations and in teaching.

This article considers leadership of integrated care as demonstrated by people with lived experience. It shares their motivation for seeking to influence services, their identities as leaders, and the use of stories within their leadership. Within the project, integrated care was interpreted as improved collaboration between professionals, services and organisations in health, social care and/or wider public services to enable people to better ‘plan my care with [those] who work together to understand me and my carer(s), allow me control, and bring together services to achieve the outcomes important to me’.^31^ ^,p.3^

## 2 METHODS

### 2.1 Design

The overall project began through discussions with the Community Advisory Board (CAB) of the Health and Social Care Leadership Centre at the University of Birmingham who wanted to better understand leadership by people with lived experience. This arose out of frustration with barriers they had encountered including tokenistic engagement opportunities and repeated consultations on the same topics with no actual progress being made. They also had a keen interest in learning of the potential solutions in other systems. CAB members had received long‐term support from health and care services and/or cared for someone with a chronic condition and/or disability. The CAB shaped the research questions, commented on the methodology, considered emerging findings and contributed to practice, policy and research implications from the project.

A qualitative design informed by narrative principles was used to gain experiences and perspectives of participants and locate these within the context of their health and care systems.^32^, ^33^, ^34^ The researcher team who undertook the interviews (R. M./N. E./C. J./V. S./W. V./A. J.) and undertook the analysis came from health and social care research, practice and/or improvement backgrounds and were based in Europe and Canada. Interviews were conducted online and lasted up to 60 min. Interviews began with an open invite for participants to share their story as to how they sought to influence integrated care. No explicit expectations were given on the nature of this story allowing participants to shape its structure and focus.^34^ Their story was followed by semistructured questions based on the following themes: positive and negative examples of engagement and co‐production, how they identified their role and what learning they would share with other people seeking to influence (see Supporting Information S1: Topic Guide).

### 2.2 Recruitment and sample

Participants were recruited by through international networks, principally those facilitated by the International Foundation for Integrated Care (IFIC), IFIC's Regional Hubs and research centres with known interest in lived experience and integrated care. Networks approached individuals who were known to have influenced change in their region or country. If the individual was interested in participating, then the research team would send further information and seek formal consent. To participate, the potential interviewee had to confirm that they had (1) accessed health and/or social care services as a patient/service recipient or as a family caregiver (2) contributed to activities relating to the review, development or oversight of health and care services, or spoken out as a campaigner or activist to make services better and (3) that these activities sought to make services more integrated (i.e. person‐centred and coordinated across professionals and services).

### 2.3 Analysis

Interviews were recorded, transcribed and anonymised. The data were analysed through the framework approach in which researchers were paired to code individual interviews and identify key themes.^35^ This process was informed by narrative principles including the dynamic between the interviewer and interviewee, how context shaped the format and content of stories, and reflecting on the emotional response of the audience (i.e., the researcher).^36^, ^37^ The research team met regularly to consider emerging themes and develop a common analytical framework which was applied to all the transcripts.^35^ Findings were shared with the CAB for comment and challenge which led to additional analysis. Issues raised included more depth on barriers faced by people from marginalised groups and practical supports provided by organisations. Research participants were invited to attend online discussions to learn of the findings and contribute their perspectives as to practice implications. Two workshops were undertaken at international conferences (International Conference on Integrated Care [ICIC] 2022 and ICIC 2023) with around 50 participants from practice, research and lived experience at each workshop. Both were structured around a brief presentation followed by a world café discussion of related themes with research team members facilitating tables and summarising insights. The ICIC 2022 discussion focussed on the background issues and research questions and ICIC 2023 on implications of findings. These validated the overall aims and conclusions of the research, helped to recruit participants, and provided good practice examples of leadership being supported.

### 2.4 Limitations

The study involved participants who were known to established research and practice networks and their insights may not fully reflect those of people with lived experience who were not accepted or choose not to engage with such networks. Despite the attempts of the research team to recruit participants from diverse backgrounds, the sample was largely women and from Western countries. Including those from different cultures and gender may have provided additional or alternative experiences.

## 3 RESULTS

Overall, 25 people with lived experience participated in the study. They were based in Europe (including United Kingdom) (10), Australasia (6), North America (5), Central America (2) and South‐East Asia (2). All had been involved in such activities for over a decade, and in some cases for much longer periods. Their roles included acting as a ‘patient advisor’ for service redesign, being a lived experience representative on organisational boards and policy groups, setting up nongovernmental organisations to provide advocacy and support and developing community programmes and networks. Their contact with services was initially based on chronic physical and/or mental health conditions and/or challenging social circumstances such as homelessness or abuse, and/or through caring for a family member who had a long‐term disability and/or degenerative conditions. Collectively participants had been able to achieve an incredible range of impacts, including changes to national policy and legislation, creation of new networks and support organisations and improving the quality of local services and professional education.

### 3.1 The origin of their leadership

The initial stories of participants had two common storylines—first, a crisis and/or chronic experience of health and social care services, and second, how participants subsequently looked to change what services are available and how these are delivered. The initial stories of contact often had the person with lived experience as the main protagonist but not always, as some were centred around the experiences of a family member, with the participant being a supporting actor:

My brother was treated very differently in the health sector to how I was treated. I found being a carer was very lonely…… You were made to feel as if you were expecting too much. (Interviewee 20)

Stories varied considerably in their scope–from time limited but intense episodes of health or social crises to ones that lasted many years and described more drawn out (but often as distressing) experiences. These could be focussed on the interaction with a particular specialism or broader engagement with a wide range of health and social care services and their lack of collaboration. The length of the stories told in the interviews varied enormously– from short accounts of 5 min to ones which lasted up to 30 min. These were structured and communicated in distinct ways—some had a more expressive approach which incorporated humour and drama, others focussed on detailing events in a more documentary style. These stories conveyed diverse, deeply personal and, in many cases, still raw experiences:

Being on the wards for six weeks total is a long time …. once I got home, I did have a long, long recovery. I had to go back for follow‐up surgery. I ended up with a couple of bowel resections, which they were able to eventually put back together after some time and healing and the use of a stoma. (Interviewee 19)

Often stories centred on instances when the participant or a family member had been treated poorly by health or care staff. This was commonly related to a lack of empathy and kindness by professionals, poor communication about plans, and being denied the opportunity to contribute to decisions about their care. Such experiences clearly did not reflect the aspirations of integrated care set out by people with lived experience.^31^ An apparent unwillingness or inability by health and care professionals to see people as equals and share their power was a common theme.

I was in this review and mentioned that he had basically splints on both legs to give him a bit more stability so that he could start to have more time in standing. And this guy was just going, ‘No, that's because of the talipes’, and I went, ‘No. If you looked at his notes he only had talipes in one leg and they've done this because he's now 18 months old and we want to get him upright.’ And this guy just basically said, ‘No, you're wrong.’ (Interviewee 17)

[The physician] looked at me and he said, ‘If you go home you'll die’ and he walked out of the room. Well, I started to cry and my wonderful husband said ‘okay don't worry I will go and talk to him’. [He] went out and said ‘could you be a little kinder to [my wife]? You know what she's been through …. to which the doctor said ‘If you don't like the way I'm treating your wife I suggest you leave’, but he went a step further, he called security … and I was left alone in emergency… (Interviewee 3)

### 3.2 Stories of change

The second storyline focussed on their personal journeys to influence health and social care services. All participants described their fundamental motivation as being to improve the experience and outcomes of other people with lived experiences or communities, and to address inequalities which meant that some members of society received poorer access and care. This was sometimes sparked through having a positive experience of receiving care, but more commonly followed negative treatment and support:

All because that day I walked into that hospital, that day with my brother and I thought, ‘It can't be like this. It just can't be’. This all started because of that. (Interviewee 20)

Many birthdays, Christmas's, like life events were held in the ICU or the Neurology Unit. And that's when I really saw a broken healthcare system where my voice did not matter, and I really thought I've got to embark on this journey to try and make a difference. (Interviewee 24)

The starting point for many was being personally invited by services to share their stories. This involved telling their personal stories at meetings, workshops or within training programmes, and arose following the participant lodging a complaint or writing to express their gratitude for the quality of care received. Others had responded to public calls for people with lived experiences to become involved in an improvement process around a service or health condition. During these initial engagements, the parameters of their stories was largely determined by services, and when these did not reflect the views of influential stakeholders were not always received positively:

After a year I went back to the CEO of that hospital and I shared all my stories about what my dad experienced, and together she and I made changes to the healthcare system. (Interviewee 3)

In this situation we're in where a disease is predominantly women and the system was being designed by men. And I tried to intervene in the design of the model of care and it wasn't taken very nicely and I got kept shutting down by the clinical lead… I wasn't being heard, I wasn't being listened to. (Interviewee 7)

I realised that most of the consumers were very affluent, middle‐class, retired, Anglo and that they had absolutely no awareness of what it is for people who have broken English to have access, that there was no equity of care. Because when you are privileged, you don't know you're privileged. (Interviewee 16)

Numerous examples were given of how directly sharing these experiences had contributed to practical changes being achieved. These included a one‐to‐one session with a chief executive leading to the creation of a space for families to have time with their dying relative, informing an improvement process which resulted in a radical overhaul of care pathways for children with a disability, and forming the basis for interprofessional education resources. Despite being asked to share very personal stories in often large audiences of highly qualified professionals and managers, no participant identified that they were given any support to prepare for the experience or indeed craft their story. Instead, we heard numerous accounts of being given limited details of what was required and then turning up at an event to be given the stage and/or microphone. While some had developed related skills in professional careers, most had no experience to draw upon and found the process to be highly intimidating:

So I did choose to share my story at what was supposed to be a presentation in front of about 50‐odd doctors. I'll never forget; I can still see it now. I turned up and they said, ‘Ah, are you ready to come in?’, and I said, ‘Oh, no, no. No, I'm not ready.’ (Interviewee 19)

When you first start off, you feel really clumsy telling your story because it doesn't flow or it doesn't address the things that they're discussing. So I've learned through trial and error to meld it a little bit, so it fits who I'm talking to. (Interviewee 20)

Having taken my clothes off for a living at one stage of my life has probably given me a little bit of that kind of confidence that you kind of need to take those first steps. (Interviewee 12)

That participants were able to overcome such daunting experiences seemed testament to their individual strength. Many connected this to the resilience that they had developed over the years in overcoming challenges relating to their health condition or social situation. The lack of support and development continued throughout people's time seeking to influence services. Where such opportunities were provided, such as educational programmes or peer mentoring, they were though highly valued. Many participants highlighted the challenges for people with lived experiences from more marginalised communities to engage in such opportunities. Alongside practical supports such as providing funding for transport and care costs and ensuring that venues were accessible, participants suggested that managers need to be more flexible and proactive in finding the spaces which worked for communities and engaging on their, rather than the service's, terms. Training and development were seen to make an important contribution to building skills and confidence:

We've just completed [a mentorship programme] with …. to mentor some younger people to step into the space. It's important. (Interviewee 20)

I've not been able to find a course, a workshop paper on the how to of being a person with lived experience leader, the nitty gritty of it. So I've been on a self‐discovery path. (Interviewee 5)

### 3.3 Storytellers or leaders?

While each participant could provide tangible examples of how they had inspired change, there was considerable divergence to self‐identifying as ‘leaders’. Some had taken on roles which explicitly included this within their title (e.g., patient leader), and others who thought that leadership was an accurate way to describe their activities despite never previously seeing themselves as such. For some, adopting the title of leader was outright rejected as it could suggest they had power or status which did not reflect their lived reality.

Yeah. I was a leader. We're like trailblazers, starting new stuff, taking it to the next level of dementia care (Interviewee 11)

I wouldn't call myself a citizen leader at all. Another one is champion; that's doing the rounds. I think ambassador used to be one that they used. I don't like any of them, particularly. (Interviewee 13)

The research team raised the term ‘citizen leader’ as this seemed to reflect the aspirations of people with lived experience taking on such activities.^15^, ^16^ Again, a proportion of participants liked the notion of citizen as for them it suggested a more inclusive constituency than only those who had accessed health and care services and the connection with rights within society. Others though strongly associated themselves as being a patient, service user or family carer, and therefore saw importance in this being included in any description:

I hadn't heard of citizen leader. I'd certainly heard of advocate, so I would've probably dodged that question if anyone asked me, ‘How do you define yourself?’ I would really struggle. I quite like the term citizen leader because it puts everyone on an equal playing field. (Interviewee 17)

I think a lot of the work that we do when we are involved as citizen leaders … and I love that term. I work really hard to incorporate the term ‘citizen’, because we're not always people with lived experience caregivers and families. (Interviewee 4)

‘I'm not sure that that type of wording, “citizen leadership,” will resonate with the rank and file’,… ‘I would say I'm a person with lived experience leader’. (Interviewee 6)

While many flaws were identified in the title of ‘citizen leader’, it is also true that no other term was seen as appropriate to all participants. A selection of other titles was shared by interviewees, including advocate, champion, advisor and partner. This reflected their differing activities, the context in which they sought to influence, and their self‐identities. The dangers of using the wrong title were also raised as these could potentially intimidate people from engaging with such activities and provide less not more clarity:

I'm not into labels. I think it would have to be used carefully. The label could put people off and they could be frightened. I mean we've gone through the same with researchers being ambassadors. It's been champions. It's been this and that. Nobody understands it. (Interviewee 2)

None of the participants explicitly titled themselves as storytellers and one saw the term as undermining due to its potential connection with fiction, when their experiences were real and personal. It was evident though that stories were core to how participants sought to influence. Their stories were entrance passes to professional and managerial forums and a powerful tool to give their views credibility, to change the perspectives of stakeholders, and to give comfort and make connection with peers:

For a long, long time getting very intimidated in a lot of those meetings and projects because every now and then, people would wheel out. ‘Well, what's your qualification?’ In the early days, your qualification was your story. (Interviewee 19)

### 3.4 Cost of sharing stories

The long‐term nature of their activities required considerable dedication on their behalf which could come at the detriment of other opportunities and life experiences, including limiting the time which they had to spend with their families and friends. There was considerable emotional toil connected with reliving difficult personal experiences. For those who could not access paid opportunities, there was also a financial cost through reduced opportunities for paid employment and career progression, and through expenses connected with their roles. Many challenges and setbacks were shared in which progress was not being made and professionals and managers were not listening to their stories for change:

When I have done hard work and have expectations for big things to happen, I involve my spare time, I involve my feelings and engagement, then I can be rather disappointed. (Interviewee 24)

This thing can become all‐consuming being so involved or wanting to be involved in things. There comes a time when you have to recognise you've got a family and you've other things or a brother who's autistic and is still needing me in his own way. (Interviewee 25)

For the past 12‐15 years as an unpaid caregiver, I'm still advocating and I'm still having a voice for the caregivers… I'm tired, burnt out and unemployed. (Interviewee 9)

Despite such frustrations, all participants could identify personal benefits. These included development of skills, knowledge and self‐confidence, meeting new people and accessing supportive networks and gaining considerable satisfaction when positive changes were implemented. A few had been able to secure paid roles which reduced the financial costs. Several described influencing services as a life‐changing process which had profoundly changed their self‐identity and enabled them to gain a deeper understanding of society. There was a ‘snowball’ effect in which the more they engaged, the more positive opportunities were presented. When organisations and partnerships had publicly acknowledged their contribution, participants were honoured by this recognition and hoped that this would demonstrate to others that they too could have an influence:

Learnt more about healthcare and how fragile and broken our systems are, but I've seen the beauty of when my voice and many other person with lived experiences and families' voices were brought in. (Interviewee 3)

I've got my own advent calendar because every time I've opened a door, there's been some new opportunity behind it. And those opportunities have grown and grown and grown. (Interviewee 6)

## 4 DISCUSSION

Activities commonly associated with citizen leadership include bridging gaps between institutions and public, mobilising people and groups to come together, and encouraging innovation in services. These, and the underlying principles such as equality, involvement and wider societal benefits reflect those of people with lived experiences seeking to influence the design and delivery of integrated care.^15^, ^16^ Similarly, their contributions can readily be associated with accepted purposes and processes of broader leadership.^7^ Therefore, while rejection of the term *citizen* by participants in this study is perhaps understandable due to potential association with more politically based activity and differences between countries, the reticence expressed by many of the term *leader* requires deeper consideration. Leadership identity construction theory^38^ suggests that self‐identification emerges from a social process in which a leader *claims* for themselves and is *granted* by others leadership identity. The common humility of people with lived experience, the perception of leadership being connected with structural power, and reluctance of managers and professionals to grant influence may explain why leadership identity was not always claimed.^13^ An added complication is that within integrated care there can be a lack of consensus regarding the purpose of engagement of people with lived experience.^39^ This is related to divergence between health and care policy and differing interpretations by professional groups.^40^, ^41^

There is of course a strong rationale which reflects the values of integrated care that people with lived experience should have agency to choose their own ‘label’.^11^ However, labels are not merely passive descriptors but actively influence the perception of others to someone's contribution and therefore the agency with which they are awarded.^42^ Symbols such as role titles both represent and shape our social identities and can enforce or challenge existing power relationships and inequalities.^41^ As ‘leader’ is generally seen as a high‐value label, declining it may diminish a person with lived experience's credibility with others and thereby restrict their scope to influence. On a more practical note, a clear definition of a person with lived experience's allocated or self‐designed role helps to outline connected skills and knowledge which in turn can enable the design and delivery of associated training and support.^39^ Not taking or being granted the title of ‘leader’ may be one reason that people with lived experiences are so rarely given access to leadership education programmes available to health care managers and professionals. It is worth noting that challenges to engagement is not down solely to the individuals—organisational cultures and professional practices provide a supportive or resistant context whatever their competence and confidence.^41^

Central to leadership influence of people with lived experience was their ability to tell stories.^14^ This reflects wider studies which emphasise the importance of storytelling to ‘create both a vision of the future and a coherent sense of the past’.^43^ ^,p.474^ While each person will have their own story and in time develop their own unique approach to telling this, there are techniques which can be taught to help personal stories take shape and impact on an audience.^44^ People with lived experience may innately reflect story telling principles such as being authentic and true, but being aware of these tools would be helpful in communicating their message.^23^ Similarly, there are story telling approaches which leaders can apply to galvanise groups not only to engage but to act.^45^ Once again, such insights, and personal development support seem largely to be absent for people with lived experience. Such opportunities could be provided on a personal basis or within a peer group. Collective approaches to develop skills and confidence have been shown to build self‐esteem and confidence, share emotional toil, build a sense of belonging and draw out common issues between members' stories.^46^ Reflecting the challenging nature of the experiences within health and social care, peer groups can provide a supportive setting for people to initially share stories and receive constructive feedback from peers on how to strengthen their impact on an audience.^46^

## 5 CONCLUSION

Despite widespread agreement as to the rationale and potential benefits of integrated care, problems persist in embedding this approach within health and care systems across the world.^47^, ^48^ One of the core enablers of achieving such change, is the creation of a common vision as to the purpose and value which resonates across different professions, sectors, and communities.^12^ Stories which articulate the urgency of change and benefits of taking common action are a powerful tool through which leaders can gain the commitment of others.^19^ Such stories are often most impactful when told by a person who has experienced related care issues as these are both authentic and emotionally engaging.^27^ All too often though integrated care programmes take an unstructured, undisciplined, and arguably unethical approach when people to share their stories.^14^ In doing so, they risk further traumatising people through asking them to relive challenging and painful experiences without putting in appropriate emotional support. Furthermore, managers and professionals often seek to place boundaries so that they, rather than the person concerned, decide on what stories should focus on and where they will be told.

Going forward, this research underlines that for integrated care to achieve its potential, more needs to be in place to support people with lived experience to feel confident in strategically influencing such developments. The emotional and practical toil of leadership must be recognised, and greater attempts made to ensure that opportunities are inclusive to those often marginalised within society. This will require sufficient investment to coordinate lived experience networks and ensure availability of practical supports such as travel, digital access and care costs. It will mean integrated care partnerships going to communities rather than expecting communities to fit in with their institutional processes and forums. Embedding leadership by people with lived experience will involve educating professionals and managers on its vital contribution. This should be designed with people with lived experience and include their stories of change. Further research is required on the effectiveness of different approaches to developing leadership by people with lived experience including the adoption of leadership identities. Perhaps we will know when integrated care has succeeded when professionals and managers are willing to grant the identity of leaders to people with lived experience and have claimed for themselves an identity of willing follower.

## AUTHOR CONTRIBUTIONS

**Robin Miller**: Conceptualisation; methodology; investigation; funding acquisition; writing—original draft; project administration; writing—review and editing; formal analysis. **Nieves Ehrenberg**: Investigation; formal analysis; writing—review and editing. **Caroline Jackson**: Methodology; data curation; investigation; formal analysis; writing—review and editing. **Viktoria Stein**: Conceptualisation; methodology; investigation; formal analysis; writing—review and editing. **Wilma Van der Vlegel‐Brouwer**: Investigation; formal analysis; writing—review and editing. **Anne Wojtak**: Conceptualisation; methodology; investigation; formal analysis; writing—review and editing.

## CONFLICT OF INTEREST STATEMENT

The authors declare no conflict of interest.

## ETHICS STATEMENT

Approval for the study was awarded by the Humanities and Social Sciences Ethical Review Committee at the University of Birmingham, United Kingdom.

## Supporting information

Supporting information.

## Data Availability

The data that support the findings of this study are available from the corresponding author upon reasonable request.
