# Supplementary material for: Just a story? Leadership, lived experience and integrated care
Source: Health Expect. 2024 May 21;27(3):e14084. doi: 10.1111/hex.14084 (PMC11109525; doi:10.1111/hex.14084)
Supplement: Supplementary file 1 — Supporting information. [file HEX-27-e14084-s001.docx]

Topic Guide

Explain the purpose of the research, and that we are taking a narrative approach based around the stories of people with lived experience who have tried to change health and social care.

Remind them that in this study, citizen leaders are people who have used their experience to speak out and help to shape integrated care services for individual benefit and for the general good.

To participate, the interviewee needs to answer yes to the following questions:

• *Have you accessed health and / or social care services as a patient / service recipient or as a family caregiver?*

*• Have you contributed to activities relating to the review, development or oversight of health and care services, or spoken out as a campaigner or activist to make services better?*

*• Have these activities been seeking to make services more integrated (i.e. person-centred and coordinated across professionals and services)?*

Themes for interviews

1. Please tell me your story – what led to you being involved in trying to shape the development or improvement of health and social care services?
2. Can you please share an experience of positive involvement? What were the circumstances and what did it entail? What / who made it positive? How did you feel? What were the impacts of these activities / experiences?
3. Can you please also share an experience of poor involvement? What were the circumstances and what did it entail? What / who made it positive? How did you feel? What were the impacts of these activities / experiences?
4. We have used the term ‘citizen leader’ in this research. How do you respond to this description of your role and contribution? Would you describe it differently and if so how?
5. If you could go back in time and talk to yourself when you started to get involved in such activities, what would be the main advice that you would share?
6. Any other stories that you would like to tell us?
